# Pain, impairment, medication use and health-related quality of life of retired professional rugby players

**DOI:** 10.17159/2078-516X/2024/v36i1a17651

**Published:** 2024-09-15

**Authors:** J Le Roux, DC Janse van Rensburg, S den Hollander, GMMJ Kerkhoffs, V Gouttebarge

**Affiliations:** 1 Amsterdam UMC location University of Amsterdam, Department of Orthopedic Surgery and Sports Medicine, Meibergdreef 9, Amsterdam, The Netherlands; 2 Section Sports Medicine, Faculty of Health Sciences, University of Pretoria, Pretoria, South Africa; 3 Division of Physiological Sciences and Health Through Physical Activity, Lifestyle and Sport (HPALS), Department of Human Biology, Faculty of Health Sciences, University of Cape Town, South Africa; 4 Academic Center for Evidence-based Sports Medicine (ACES), Amsterdam, The Netherlands; 5 Amsterdam Collaboration on Health & Safety in Sports (ACHSS), IOC Research Center of Excellence, Amsterdam, The Netherlands; 6 Amsterdam Movement Sciences, Sports, Musculoskeletal Health, Ageing & Vitality, Amsterdam, The Netherlands

**Keywords:** Rugby, retired, pain, impairment, mental health

## Abstract

**Background:**

Rugby is a physically demanding sport with a high injury rate. Professional male rugby players have a notably greater risk of sustaining injuries that require hospitalisation or surgery than male athletes from non-contact sports. Retired elite male rugby players experience physical and mental health conditions as well as varying levels of pain, negatively impacting their quality of life. Retired rugby players could use medication or substances as a coping mechanism to deal with chronic pain and a lower quality of life. However, research is scarce on how retired rugby players manage pain and how this affects their quality of life.

**Objectives:**

This study aimed to understand joint pain and impairment, mental and physical quality of life, and pain medication use in retired professional male rugby players.

**Methods:**

A cross-sectional study was conducted using a questionnaire completed by retired professional male rugby players. Joint pain and impairment were explored through three questions, health-related quality of life was assessed through the PROMIS-GH, and medication use was explored through 12 questions.

**Results:**

Retired rugby players (N=142) reported higher scores than matched controls (N=49) for joint pain and impairment, including significantly higher scores for joint impairments for activities of daily living (*p*=0.047). The global mental health scores of retired rugby players were significantly lower compared to matched controls (p=0.043) and the global physical health scores were also lower in retired rugby players. Most retired rugby players reported not using prescription pain medication (75%) or over-the-counter pain medication (56%).

**Conclusion:**

Professional rugby careers have a considerable impact on the joint health and overall well-being of retired players, resulting in unique challenges. The findings of this study emphasise the need for specific after-career support for challenges faced by retired rugby players.

Rugby union (hereafter referred to as rugby) is a physically demanding team sport where players engage in powerful collisions and strategic gameplay. Rugby has a high injury rate compared to other team sports, and professional male rugby players have a notably greater risk of sustaining injuries that require hospitalisation or surgery than male athletes from non-contact sports.[1, 2] Retired elite male rugby players experience physical and mental health conditions, negatively impacting their quality of life.[3, 4] A scoping review showed that 51–60% of retired elite rugby players have osteoarthritis, likely to cause varying pain levels in previously injured areas.[4] Joint pain is the second most common type of pain (after back pain) in retired elite male rugby professionals.[3]

A 2020 systematic review found that using opioids is common among athletes, including professional athletes.[5] In American football, a highspeed collision sport considered a distant 'cousin' of rugby, retired players use opioids at three times the rate of the general population and misuse of opioids was also predicated by, amongst other factors, significant pain.[6] The prevalence of hazardous alcohol use in retired rugby players is reported to be 36–37%.[4] It is essential to understand how retired rugby players manage pain and how frequently they use pain medication, as uncontrolled use could lead to various complications. The use of pain medications has been positively linked with psychological distress and an increased risk of suicide, while the misuse of prescription pain medication has been associated with a higher resting heart rate, cognitive impairments, and organ damage.[7–10] One possibility of how retired rugby players could deal with chronic pain and a lower quality of life is through using medication or substances as a coping mechanism. However, research is scarce on how retired rugby players manage pain and how this affects their quality of life.

Therefore, this study aimed to understand joint pain and impairment, mental and physical quality of life, and pain medication use in retired professional male rugby players. This study achieved this through the following objectives: 1) describe the level of joint pain and impairment in retired professional male rugby players and compare to matched controls from the participants’ social circle from a non-elite sporting background, 2) describe the mental and physical quality of life (QoL) of retired rugby players and compare to matched controls from the participants’ social circle from a non-elite sporting background, 3) explore the association between levels of joint pain and mental and physical quality of life of retired rugby players, and 4) describe the medication use of retired rugby players and assess the association with the level of joint pain.

## Methods

### Study design

A cross-sectional study using a questionnaire was conducted. The Strengthening the Reporting of Observational Studies in Epidemiology (STROBE) statement was applied to improve the reporting standard.[11] The Medical Ethics Review Committee of the Amsterdam University Medical Centers, location Academic Medical Center (W16_366#16.431; Amsterdam, the Netherlands) granted ethical approval for the study. The study was conducted according to the Declaration of Helsinki (2013).

### Participants and matched controls

The population for this study comprised retired professional male rugby players recruited by the International Rugby Players (IRP). The inclusion criteria were: a) retired professional male rugby player, b) age 50 or below, c) male, and d) literate in English or French. In our study, a retired professional rugby player is someone who a) before retirement, participated in rugby in the highest or second-highest national division, b) regularly took part in exercise and training activities to enhance their performance, and c) spent a significant amount of time on all or most days training or competing. Participants were also requested to utilise their social networks to obtain one control from their peer group and without an elite sporting background matched for gender, age, height, and body weight. Sample size calculation concerning the presence of pain indicated that 138 participants were needed (power of 80%, confidence interval of 95%, absolute precision of 5%) under the assumption of an anticipated population proportion of 10%.[12] As a response rate of approximately 50% was expected, an attempt was made to reach at least 300 potential participants.

### Outcome measurements

#### Pain and impairment

Pain in any joint was explored through a single question (‘Over the past week, on average, how would you rate your joint pain?’) scored on an 11-point scale, from “No pain” (0) to “Worst pain possible” (10). Impairment was explored through two questions: one about impairment in daily activities (housework, washing, dressing, lifting, reading, and driving) and one about impairment in recreational, social, and family activities (e.g., ‘Over the past week, how much has your joint pain interfered with your ability to take part in recreational, social, and family activities?’). These two questions were scored on an 11-point scale, from “No interference” (0) to “Unable to carry out activity” (10).

#### Health-related quality of life

The Patient-Reported Outcomes Measurement Information System Global Health short form (PROMIS-GH) was used to assess multiple domains related to health-related quality of life, such as health, functioning, pain, social activities, and fatigue.[13] The PROMIS-GH has been validated in several populations and languages, including English and French (for detailed information, see www.nihpromis.org).[13] Based on ten items, each measured on a 5-point scale (from 1 to 5) and subsequently converted, the Global Physical Health and Global Mental Health scores were calculated.[13] The Global Physical Health score was calculated by adding the scores of items 3, 7, 9, and 10 of the quality of life questions on the questionnaire to obtain a subscale score out of 20. The questions relate to physical health, ability to carry out social activities and roles, fatigue, and pain. The Global Mental Health score was calculated by adding the scores of items 2, 4, 5, and 8 to obtain a subscale score out of 20. The questions relate to quality of life, mental health, satisfaction with social activities and relationships, and being bothered by emotional problems. A higher score indicated a better quality of life (PROMIS, see www.nihpromis.org).

#### Medication and substance use

Medication and substance use were explored through 12 questions. Medications related to analgesics (prescription), analgesics (over-the-counter), allergy medications, asthma medications, insulin, sedatives (prescription), sedatives (over-the-counter), antidepressants, and diuretics were explored. All questions were measured on a 5-point scale, from “I do not use” (0) to “I use daily”(4).

### Procedures

An anonymous electronic questionnaire was compiled in English and French (LimeSurvey Professional Lim GmbH). The questionnaire included the following descriptive variables: age, height, body weight, duration of professional rugby career, main level of play, position, duration of retirement, nature of retirement, and employment status. The IRP contacted potential participants and provided them with all necessary information about the study. Participants who were willing to participate provided informed consent and completed the electronic questionnaire. Participants were requested to complete the questionnaire within two weeks and were sent reminders about completing the questionnaire after two weeks and four weeks. The responses were coded and anonymised to ensure privacy and confidentiality. The completed electronic questionnaires were saved automatically on a secured electronic server, and only the principal researcher could access the server. Players participated voluntarily in the study and were not rewarded for their participation.

### Statistical analyses

The statistical software IBM SPSS 29.0.1.0 for Apple Mac was used for all data analyses and the level of significance was set at p≤0.05. Descriptive analyses (mean and standard deviation (SD) were performed for all variables in the study separately for retired rugby players and matched controls from the participants’ social network without an elite sporting background.

The mean scores out of ten for joint pain, joint pain interference with daily activities, and joint pain interference with social activities for the population of this study were compared to the matched control group’s scores using the Mann-Whitney U test. This test was used because the data were not normally distributed, and the Mann-Whitney U test can be used to compare unequal sample sizes.

The mean scores out of 20 for global physical health and global mental health for the two populations were compared, also using the Mann-Whitney U test as the normality assumption was also violated in the data.

To explore the association between the levels of joint pain and mental and physical quality of life of retired rugby players, a Spearman Correlation was performed as the data did not meet the requirements for the parametric tests. The Dancey and Reidy’s (2004) correlation categorisation was used to describe the strength of the correlations, which states correlation coefficient: 0.1–0.39=weak correlation, 0.4–0.69=moderate correlation, 0.7–0.9=strong correlation, 1=perfect correlation.[14]

The frequency of medication use was provided for prescription analgesics and over-the-counter analgesics. For prescribed analgesics and over-the-counter analgesics, the ordinal categorical variable was converted to a numeric variable (Daily=4, Weekly=3, Monthly=2, Less often =1, Do not use=0) to explore the association between the level of joint pain and frequency of pain medication use. Subsequently, a Spearman correlation was performed for both variables and the Dancey and Reidy’s (2004) correlation categorisation was used to describe the strength of the correlation.[14]

## Results

### Participants

From 326 rugby players contacted, 146 gave informed consent and 142 fully completed the questionnaire (response rate of 44%), while 52 matched controls were enrolled in the study and 49 fully completed the questionnaire. The mean age of the retired players was 39.61 years (SD=5.88), and the mean duration of their careers was 10.66 years (SD=4.24). On average, the players had been retired for 8.18 years (SD=5.81). Table 1 presents the characteristics of the participants and matched controls.

### Joint pain and impairment

The retired rugby players reported higher scores than the matched controls for all three domains of joint pain (scores out of ten), as indicated in Table 1. Their score for joint impairments for activities of daily living in the last week (χ̄=1.2) was significantly higher than the matched control group (χ̄=0.7) (*U*=3109.00, *z*=−1.99, *p*=0.047).

### Mental and physical quality of life

The global mental and physical health scores out of 20 were lower in the retired rugby players group than in the matched control group (Table 1). The global mental health score of the retired rugby players (χ̄=13.63) was significantly lower compared to the matched control group (χ̄=14.71) (U=3083.00, z=−2.07, p=0.043).

### Association between levels of joint pain and mental and physical quality of life

There was a weak negative correlation between joint pain and the global physical health score (correlation coefficient −0.30; p<0.001) and a weak positive correlation between joint pain and the global mental health score (correlation coefficient 0.14; p=0.082). More information on the correlation between joint pain and global physical and global mental health is provided in Table 2.

### Medication use and its association with the level of joint pain

**Fig. 1:**
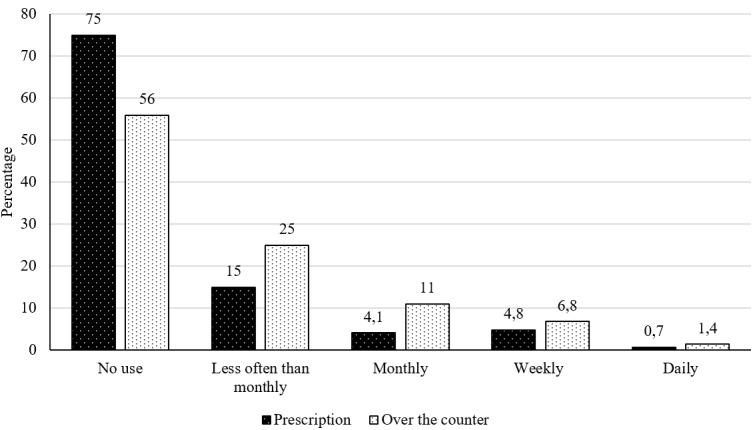
Percentage of retired rugby players who use pain medication

Fewer than a quarter of the retired rugby players (25%) reported using any prescription pain medication, while 15% reported using less than monthly. Fewer than half of the retired rugby players reported using any over-the-counter pain medication. Meanwhile, 25% reported using over-the-counter pain medication less than monthly. Figure 1 presents the pain medication used by retired rugby players.

Regarding other medication use, 19% of the retired rugby players reported using allergy medication, 10% reported using asthma medication, and only one participant reported using insulin. None of the retired rugby players uses diuretics, 12% use over-the-counter sleeping tablets, 9.6% use prescription sleeping tablets, and only 3% of the participants use antidepressants.

There was a weak positive correlation between joint pain in the past week and the use of over-the-counter and prescription pain medication use in retired rugby players. The correlation of the prescription medication use was slightly stronger (moderate) (correlation coefficient 0.40; p<0.001) than the use of over-the-counter pain medication (correlation coefficient 0.26; p < 0.001). More information on the correlation between joint pain and the use of pain medication is provided in Table 2.

## Discussion

This study aimed to describe joint pain and impairment, mental and physical quality of life, and pain medication use in retired professional male rugby players. The retired rugby players reported significantly higher levels of joint impairment for activities of daily living compared to the matched control group. In comparison, their levels of joint pain and joint impairments for recreational and social activities were also higher. The retired rugby player population’s global mental and physical health levels were both lower compared to the matched controls, with a significant difference in the global mental health score. A weak negative correlation was observed between joint pain and the global physical health score, and a weak positive correlation between joint pain and the global mental health score. Most of the retired rugby players reported that they do not use pain medication, and there was a moderate correlation between the retired rugby players experiencing joint pain in the past week and prescription medication use.

### Joint pain and impairment

Joint pain is a common and complicated occurrence for retired elite rugby players.[3] Pain is subjective, and individuals form their understanding of pain throughout their lives.[15] From a young age, rugby players are compelled to endure pain as part of their sport, and this pattern persists throughout their careers.[16] For example, more than half of rugby players continue playing rugby despite describing shoulder dysfunction, indicating their ability to continue playing and training unimpeded despite pain and injuries.[17] There appears to be a normalisation of “suffering in silence”, meaning players could be compelled to endure pain and pretend to be unaffected.[16] This could be a reason why our study found that retired professional rugby players had higher levels of joint pain and impairment for recreational and social activities compared to their peers from a non-elite sporting background and a significantly higher level of joint impairments for activities of daily living. Rugby players’ ability to persist with activities despite experiencing pain could continue after their careers, leading players to endure pain when participating in social and recreational activities similar to playing rugby and accept that it is part of it. However, when it comes to the perceived impairment, it seems to vary and with activities of daily living, the pain appears to be more pronounced.

### Mental and physical quality of life

Research on mental health in retired athletes, including retired rugby players, has increased in recent years, with multiple studies showing that retired rugby players have a higher prevalence of mental health symptoms than the general population.[18–21] In particular, the prevalence of depression and anxiety symptoms was as high as 49% in retired elite rugby players.[4] At the same time, on average, half of retired elite rugby players were found to be engaging in hazardous alcohol use.[4, 21] Our study confirms the mental health difficulties experienced by retired elite rugby players as it found that retired elite rugby players' global mental health was significantly lower than that of their peers. The challenges that elite rugby players experience during and after their careers require a better understanding of how this affects their long-term mental health. Even feeling compelled to endure pain over the long term could result in poorer mental health or being forced to develop negative coping strategies. This remains an important area that needs further investigation.

### Medication use and its association with the level of joint pain

There are many anecdotal reports on the prevalence of pain medication use in professional rugby.[22, 23] However, scientific research on this topic is scarce. In American football, retired players use three times the amount of opioids compared to the general population, often as a result of pain.[6] Our study found that most retired rugby players do not use prescribed or over-the-counter pain medication. The rate of pain medication use also appears to be lower than average as compared to adult men of similar ages. A study showed that 50% of men in France between the ages of 35 and 44 use any form of analgesics.[24]

The lower use of pain medication appears to be in contrast to the pain experienced by this population, which could again point to the fact that retired players can cope with their pain without medication or perhaps suggest they use other methods to deal with their pain, such as alcohol use which is often prevalent in this population.[18, 20]

### Limitations

One of the limitations of this study is the reliance on only self-report questionnaires which could threaten the internal validity of the study. Self-report questionnaires are often easy to administer, meaning data can be obtained from a wide range of participants, but the study is susceptible to response bias. However, it remains a preferred way of collecting data from a wide range of participants. Another limitation is that the study's findings may not be generalisable as the respondents who decided to participate might have been particularly affected or interested in the topics under investigation. Despite the anonymity of the questionnaires, retired rugby players might have been hesitant to accurately report their medication use due to the desire to appear unaffected by pain. This study also did not consider any post-rugby career factors in its findings. A further limitation of this study is the difference in sample size of the retired professional rugby players group and the matched controls which could limit the generalisability of the findings. Each participant was requested to recruit a matched control, however, not every retired professional rugby player complied, resulting in discrepancies in sample sizes between groups.

### Implications for research

More research is required to better understand the unique challenges experienced by male and female retired elite rugby players. Exploring the long-term experiences in their mental and physical health will provide a better understanding of these challenges and inform how to support them better. In particular, there is a need to understand the different aspects of pain perceptions and management and its impact on retired rugby players. Qualitative research allowing retired players to share more insights into their unique experiences and challenges in their retirement years could contribute.

The mental health of current and retired elite rugby players has received increased attention in recent years, but this remains an area of concern for this population (as for former elite athletes from other sports). Further investigation is required to better understand the unique psychological challenges of transitioning out of professional rugby and the different challenges retired players experience at varying times during their retirement. Assessing players at different time periods in their retirement years is an option to create more support for these players.

There is also a critical need to understand pain management through medication and substance use, such as alcohol, in retired elite rugby players, as there is little research available in this area. Examining how retired rugby players use pain medication can offer guidance on managing their pain best and how health practitioners and decision-makers can support this group.

### Implications for practice

Based on the findings from this paper, retired elite rugby players need more support. This population experiences unique challenges and requires tailored support during their post-career life. One of the support measures that could be of added value to retired elite rugby players is the After Rugby Career Consultation which was created to support retired professional rugby players’ long-term health and quality of life.[25]

## Conclusion

The findings of our study emphasise the considerable impact professional rugby careers have on the joint health and overall well-being of retired players. The elevated levels of joint impairment, particularly for activities of daily living, as well as the lower global mental and physical health scores compared to their peers from a non-elite sporting background, highlight the unique challenges retired professional rugby players experience. The findings emphasise the need for specific after-career support for the challenges faced by retired rugby players.

## Figures and Tables

**Table 1:** Characteristics and outcome measures of retired rugby players and matched controls

	Retired rugby players (N=142)	Matched controls (N=49)	p-value

Age (years)	39.6 ± 5.9	39.2 ± 6.5	0.684
Height (cm)	185.8 ± 8.0	184.8 ± 6.2	0.473
Weight (kg)	102.3 ± 15.6	98.8 ± 14.2	0.183
Duration of rugby career (years)	10.7 ± 4.2		
Duration of retirement (years)	8.2 ± 5.8		
Joint pain in past week (mean score out of ten)	1.3 ± 2.0	1.1 ± 2.1	0.358
Joint impairments for activities of daily living in the past week (mean score out of ten)	1.2 ± 1.5	0.7 ± 1.0	0.047*
Joint impairments for recreational and social activities in the past week (mean score out of ten)	0.9 ± 1.4	0.6 ± 0.9	0.448
Global physical health (mean score out of 20)	15.7 ± 2.6	16.1 ± 2.6	0.363
Global mental health (mean score out of 20)	13.7 ± 2.5	14.7 ± 2.7	0.043*

Data expressed as mean ± standard deviation.

* indicates p<0.05.

**Table 2:** Correlation between the level of joint pain in the past week, mental and physical quality of life, and medication use of retired rugby players (N=142)

	Correlation coefficient	p-value	Correlation strength

Correlation between level of joint pain and global physical health	−0.30	<0.001	Weak*
Correlation between level of joint pain and global mental health	0.014	0.082	Weak*
Correlation between level of joint pain and use of prescription pain medication	0.40	<0.001	Moderate*
Correlation between level of joint pain and use of over-the-counter pain medication	0.26	0.002	Weak*

* According to Dancey and Reidy’s (2004) correlation categorization (0.1–0.39=weak correlation, 0.4–0.69=moderate correlation, 0.7–0.9=strong correlation, 1=perfect correlation).[14]
